# Pan-NLRome Analysis of Cultivated Tomato and Wild Solanum Relatives Reveals an Open Immune Repertoire Dominated by Spatially Dispersed Homologs

**DOI:** 10.3390/genes17080864

**Published:** 2026-07-24

**Authors:** Shibo Meng, Jiajun Zhu, Enmei Hu, Jia Liu, Yuan Cheng, Meiying Ruan, Chenxu Liu, Qingjing Ye, Rongqing Wang, Zhuping Yao, Zhimiao Li, Guozhi Zhou, Hongjian Wan, Yougen Chen

**Affiliations:** 1 College of Horticulture, Anhui Agricultural University, Hefei 230036, China; 24720187@stu.ahau.edu.cn (S.M.); 24721214@stu.ahau.edu.cn (J.Z.); 2 State Key Laboratory for Quality and Safety of Agro-Products, Institute of Vegetables, Zhejiang Academy of Agricultural Sciences, Hangzhou 310021, China; liu-l-jia@163.com (J.L.); chengyuan@zaas.ac.cn (Y.C.); ruanmy@zaas.ac.cn (M.R.); liuchenxu@zaas.ac.cn (C.L.); yeqj@zaas.ac.cn (Q.Y.); wangrq@zaas.ac.cn (R.W.); yaozp@zaas.ac.cn (Z.Y.); zhimiaoli@zaas.ac.cn (Z.L.); zhougz@zaas.ac.cn (G.Z.); 3 Department of Agronomy and Horticulture, Jiangsu Vocational College of Agriculture and Forestry, Jurong 212400, China; xfx202202@jsafc.edu.cn

**Keywords:** NLR, pan-NLRome, disease resistance, plant immunity, comparative genomics

## Abstract

**Background/Objectives:** Nucleotide-binding leucine-rich repeat (NLR) proteins are major intracellular immune receptors involved in effector-triggered immunity. However, the evolutionary diversity and genomic organization of NLR repertoires remain incompletely characterized in Solanaceae crops and their wild relatives. This study aimed to investigate the pan-NLRome landscape and evolutionary patterns of tomato and related species. **Methods:** A comparative pan-NLRome analysis was performed across five angiosperms, including cultivated tomato (*Solanum lycopersicum*) and four related species (*Solanum chilense*, *Solanum lycopersicoides*, *Solanum pimpinellifolium*, and *Arabidopsis thaliana*). NLR genes were identified using an integrated HMMER- and BLASTp-based pipeline, followed by chromosome anchoring, orthogroup (OG) classification, phylogenetic analysis, spatial organization analysis, and evaluation of associations with long terminal repeat (LTR) retrotransposons. **Results:** A total of 1566 chromosome-anchored NLR genes were assigned to 150 OGs. Core OGs represented 25.3% of total OG diversity but contained a large proportion of NLR genes. Rarefaction analysis indicated continuous accumulation of novel OGs with increasing species sampling, supporting an open pan-NLRome structure. Phylogenetic analysis identified 18 NLR subfamilies, with SF_03 and SF_01 together accounting for approximately 79% of NLR genes. Dispersed homologs represented the predominant spatial arrangement pattern, accounting for 85.1% of NLR gene pairs across Solanaceae species. **Conclusions:** This study provides a comparative genomic framework for understanding NLR diversity and evolution in tomato and related Solanum species, highlighting the dynamic expansion and spatial organization of plant immune receptor repertoires and providing valuable resources for resistance gene discovery.

## 1. Introduction

Plants have evolved a sophisticated, multi-layered innate immune system to defend against diverse pathogen invasions [1]. The first layer of defense is pattern-triggered immunity (PTI), which is activated when cell-surface pattern recognition receptors (PRRs) detect conserved pathogen-associated molecular patterns (PAMPs). To overcome this barrier, successful pathogens deliver effector proteins into host cells to suppress PTI; however, these effectors can in turn be recognized by intracellular immune receptors, eliciting a more robust defense response known as effector-triggered immunity (ETI) [1,2]. This reciprocal interplay between PTI and ETI is elegantly captured by the “ZigZag” [2]. However, accumulating evidence has demonstrated that PTI and ETI are not strictly independent or sequential immune layers, but rather exhibit extensive mechanistic crosstalk and synergistic interactions. Therefore, the ZigZag model should be viewed as an evolving conceptual framework that continues to be refined as our understanding of plant immune signaling advances [2].

The primary intracellular receptors mediating ETI belong to the NLR protein superfamily [1,3]. These proteins have a conserved ternary structure: the core is an NB-ARC domain that acts as a molecular switch, the C-terminal is a leucine-rich repeat (LRR) domain that participates in self-regulation and pathogen recognition, and the N-terminal is a variable domain, which is divided into three subclasses—TNL (containing TIR domain), CNL (containing coiled-coil domain), and RNL (containing RPW8-like domain) [3,4]. Upon activation, the NLR protein will undergo oligomerization to form a poly-resistant body complex: CNL proteins such as ZAR1 can form calcium permeability channels and trigger calcium influx; TNL proteins catalyze the hydrolysis of NAD to produce signal metabolites such as cyclic ADP ribose, which jointly initiate immune signal transduction and induce local hypersensitive cell death [5,6,7,8,9]. In addition, some NLR proteins also carry integration domains (NLR-IDs), which can be used as a molecular bait for pathogen effectors, further enriching the diversity and recognition specificity of the NLR immune network [3,4].

The NLR gene family ranks among the most dynamic in plant genomes: rapid birth-and-death dynamics—tandem duplication, segmental duplication, and transposable element (TE)-mediated rearrangements—drive NLR copy number and composition to vary substantially across and within species, reflecting an ongoing co-evolutionary arms race with rapidly diversifying pathogen effectors [2,9,10]. This dynamism has long challenged the comprehensive characterization of NLR gene pools using any single reference genome [4].

The pan-genome concept, originally developed in prokaryotic genomics, has been increasingly applied to crop species to capture the full spectrum of genomic variation beyond what a single reference assembly can represent [11,12,13]. Extending this framework to the NLR gene family has given rise to the Pan-NLRome, which encompasses the complete NLR repertoire within a defined set of genotypes or species, including both conserved core components and variable dispensable components [4,14]. Pan-NLRome analysis of *A. thaliana* demonstrated that most NLR orthologous groups exhibit low frequency and are species- or genotype-specific, indicating that the pan-NLR space has an open structure that continues to expand as more genotypes are sampled [4]. In tomato (*S. lycopersicum*), large-scale pan-genome and graph-based pangenome studies have demonstrated that structural variations—including gene presence/absence variations—make important contributions to phenotypic diversity and agronomic traits [15,16]. Nevertheless, systematic NLR-specific pan-genome characterization spanning multiple species within the Solanaceae family remains limited.

Solanaceae encompasses numerous economically important crop species, including tomato, potato (*Solanum tuberosum*), pepper (*Capsicum annuum*), and eggplant (*Solanum melongena*), many of which suffer from devastating bacterial, fungal, oomycete, and viral diseases [1,3]. Previous single-species studies have characterized NLR gene pools within Solanaceae, revealing conserved patterns of tandem duplication, gene clustering, and spatial distribution; in pepper, for example, dispersed NLR homologs account for 61.5% of the repertoire, with tandem and proximal arrangements notably more prevalent than in many other species [15,17]. Despite these advances, a comprehensive cross-species Pan-NLRome analysis in Solanaceae—addressing OG conservation and divergence, phylogenetic subfamily structure, and genome-wide spatial organization—has yet to be conducted.

Transposable elements, particularly LTR retrotransposons, constitute a substantial proportion of plant genomes and have been implicated in the amplification, diversification, and spatial reshuffling of NLR gene families [9,10,18]. Preferential co-localization of LTR retrotransposons with NLR gene clusters has been reported across multiple species [10], suggesting that TE-mediated rearrangements may underlie the characteristically dispersed distribution of NLR gene pools. However, the statistical robustness and evolutionary significance of this spatial association between LTR retrotransposons and NLR genes in the cultivated tomato genome have not been rigorously assessed.

In this study, we performed a comparative Pan-NLRome analysis across five angiosperm species, with cultivated tomato as the focal reference. Specifically, we: (1) identified and annotated the genome-wide NLR repertoire using an integrated pipeline combining HMMER and BLASTp; (2) assigned NLR genes to orthologous groups and characterized the core–shell–cloud structure of the tomato pan-NLRome; (3) reconstructed the phylogenetic relationships among NLR lineages and delineated subfamily boundaries; (4) quantified spatial organization patterns of NLR homologs and compared them with published data from pepper; and (5) systematically evaluated the spatial association between NLR genes and LTR retrotransposons in the cultivated tomato genome. Collectively, these analyses provide a comprehensive genomic framework for understanding NLR diversity, evolution, and spatial organization in Solanaceae and lay the groundwork for future disease resistance gene discovery and crop improvement.

## 2. Materials and Methods

### 2.1. Genomic Datasets and Gene Annotation Resources

Genome assembly sequences and corresponding gene annotation files (GFF3 format) were obtained for eight representative angiosperm species: cultivated tomato (*S. lycopersicum* cv. Heinz 1706, assembly SL4.0, annotation ITAG4.0 [19]), *S. chilense*, *S. lycopersicoides* LA2951, *S. pimpinellifolium* LA1589, *Solanum habrochaites* LA1353, eggplant (*S. melongena*), potato (*S. tuberosum*), and *A. thaliana* (TAIR10 [20]). Detailed accession information and data sources are provided in the Data Availability Statement. During preliminary processing, the annotation structures of *S. habrochaites*, *S. melongena*, and *S. tuberosum* were found to be incompatible with downstream NLR gene extraction and chromosome anchoring pipelines, owing to inconsistent gene identifiers and incomplete positional information. These three species were therefore excluded from comparative genomic analyses. All subsequent Pan-NLRome analyses were conducted on the remaining five species: *S. lycopersicum*, *S. chilense*, *S. lycopersicoides*, *S. pimpinellifolium*, and *A. thaliana*. The assembly quality of the analyzed genomes was heterogeneous. The *S. lycopersicum* SL4.0 reference genome represents a chromosome-scale assembly generated using long-read sequencing technologies, whereas several other available assemblies exhibit lower contiguity. Because repetitive genomic regions, including pericentromeric regions enriched in NLR clusters, are often difficult to resolve in fragmented assemblies, differences in assembly continuity may influence the completeness of NLR annotation. Therefore, assembly quality was considered together with annotation compatibility during species selection. Although preliminary NLR candidates were identified from all available datasets, only chromosome-anchored species with reliable annotations were retained for downstream comparative analyses.

### 2.2. Genome-Wide Identification of NLR Genes

To identify candidate NLR genes, protein sequences from each species were screened using HMMER v3.3.2 [17] against the NB-ARC domain profile (PF00931, from the Pfam database [16]), with an E-value threshold of ≤1 × 10^−5^. Candidate proteins were further validated using BLASTp v2.5.0 (NCBI, Bethesda, MD, USA) against the predicted proteome of the corresponding species [21]. Sequences were retained when they showed the best similarity to annotated NLR-related proteins with an E-value ≤ 1 × 10^−5^, sequence similarity ≥ 75%, and alignment length ≥ 300 amino acids. The resulting NLR gene sets were used for subsequent comparative genomic and evolutionary analyses.

### 2.3. Chromosomal Distribution, Gene Density, and Synteny Analysis

The chromosomal distribution of NLR genes in cultivated tomato was visualized using Circos v0.69 [22]. Based on physical coordinates extracted from the SL4.0/ITAG4.0 annotation, NLR genes were mapped onto the 12 tomato chromosomes. Gene density was calculated using a 1 Mb sliding window with a step size of 100 kb. Syntenic relationships among NLR genes were identified using MCScanX v1.0.0 [23], retaining only collinear blocks containing at least one NLR gene for downstream visualization. A collinearity network was subsequently constructed, in which nodes represent individual NLR genes, and edges connect genes located within the same syntenic blocks. Network visualization was performed using Cytoscape v3.10 [24] with a force-directed layout algorithm. Using this pipeline, a total of 1303 NLR-related collinear gene pairs were identified in the cultivated tomato genome.

### 2.4. OG Inference and Pan-NLRome Classification

The pan-NLRome was defined here as the complete collection of NLR homologous gene clusters identified across the five study species, representing a cross-species comparative framework rather than a within-species pan-genome. Orthologous relationships among NLR genes were inferred using OrthoFinder v2.5.4 [18] with default parameters. OGs were classified into three categories based on their species distribution:

• Core OGs: present in all five species;
• Shell OGs: present in 2–4 species;
• Cloud OGs: species-specific, present in only one species.

The proportions of core, shell, and cloud OGs were calculated based on both OG count and total gene content. To evaluate pan-NLRome openness, the cumulative number of OGs and the number of novel OGs introduced by each successive species were recorded across 100 randomized species-order permutations. For each permutation, the mean and 95% confidence interval of the cumulative OG count were calculated and plotted as a function of species number.

### 2.5. Phylogenetic Analysis and Subfamily Classification

For each OG, the longest protein sequence was selected as the representative. Multiple sequence alignment of representative proteins was performed using MAFFT v7.525 [25] with the --auto strategy. Poorly aligned regions were trimmed with trimAl v1.4 [26] using the -automated1 parameter. Maximum likelihood phylogenetic trees were then constructed using FastTree v2.1.11 [27] under the WAG+GAMMA substitution model. Subfamily boundaries were delineated by integrating phylogenetic topology, branch support values (local support ≥ 0.90), and OG clustering patterns, followed by manual curation. This procedure partitioned the 150 OGs into 18 subfamilies (SF_01–SF_18). Phylogenetic trees and associated metadata were visualized using iTOL v6.0 [28], with annotation layers indicating core/shell/cloud classification, OG size, and species distribution.

### 2.6. Spatial Organization Patterns of NLR Homologs

To characterize the genomic organization of homologous NLR genes, gene pairs belonging to the same OG were classified into four spatial arrangement categories based on chromosomal proximity:

• Tandem: inter-gene distance ≤ 200 kb and ≤10 intervening genes;
• Proximal: inter-gene distance ≤ 200 kb but >10 intervening genes;
• Dispersed: genes located on different chromosomes or separated by >200 kb;
• Singleton: genes for which no homologous partner within the same OG could be detected under the above criteria.

The proportions of each organizational pattern were calculated across the five species. For cross-family comparisons, Arabidopsis was analyzed separately from the four Solanaceae species. Results were compared with the NLR organization patterns reported for pepper (*C. annuum*) by Seo et al. [15].

### 2.7. Association Between LTR Retrotransposons and NLR Genes

To investigate the potential association between transposable elements and NLR genomic organization, full-length LTR retrotransposons (LTR-RTs) in the cultivated tomato genome were identified using a combined ab initio prediction approach. LTR_FINDER v3.0.7 [29] and LTRharvest [30] were applied independently to predict candidate LTR-RTs, and the resulting predictions were subsequently merged, de-duplicated, and filtered for structural integrity using LTR_retriever v3.0.6 [31], yielding 3064 high-confidence, full-length LTR-RTs. For each NLR gene, the presence of LTR-RTs within flanking genomic windows of ±2, ±5, ±10, ±20, ±50, and ±100 kb relative to the transcription start site was examined. Enrichment significance was assessed using Fisher’s exact test, with background rates estimated from 1000 random samplings of genomic regions of equivalent size to correct for sampling variance. Additionally, the distance from each NLR gene to its nearest full-length LTR-RT was calculated. Differences in these distances between core NLR genes and non-core (Shell + Cloud) NLR genes were assessed using the Mann–Whitney U test.

### 2.8. Statistical Analysis and Visualization

All statistical analyses were conducted in Python v3.9 (Python Software Foundation, Wilmington, DE, USA) using the scipy v1.11 and statsmodels v0.14 libraries. Fisher’s exact test was implemented via scipy.stats.fisher_exact() and the Mann–Whitney U test via scipy.stats.mannwhitneyu(). Data visualization was performed using matplotlib v3.8 and seaborn v0.13. Unless otherwise stated, the significance threshold was set at *p* < 0.05, with multiple testing correction applied using the Benjamini–Hochberg procedure [32] where appropriate.

## 3. Results

### 3.1. Genome-Wide NLR Gene Identification Across Five Angiosperms

A total of 2979 candidate NLR genes were initially identified from eight available Solanaceae and reference genomes. However, due to differences in genome assembly continuity and annotation compatibility, reliable chromosomal anchoring could not be achieved for *S. habrochaites*, *S. melongena*, and *S. tuberosum*. Therefore, these three species were excluded from all downstream comparative analyses. The final pan-NLRome analysis was conducted using five chromosome-anchored species with comparable genome annotation quality (Table 1).

### 3.2. Chromosomal Distribution and Syntenic Organization of Tomato NLR Genes

**Figure 1:**
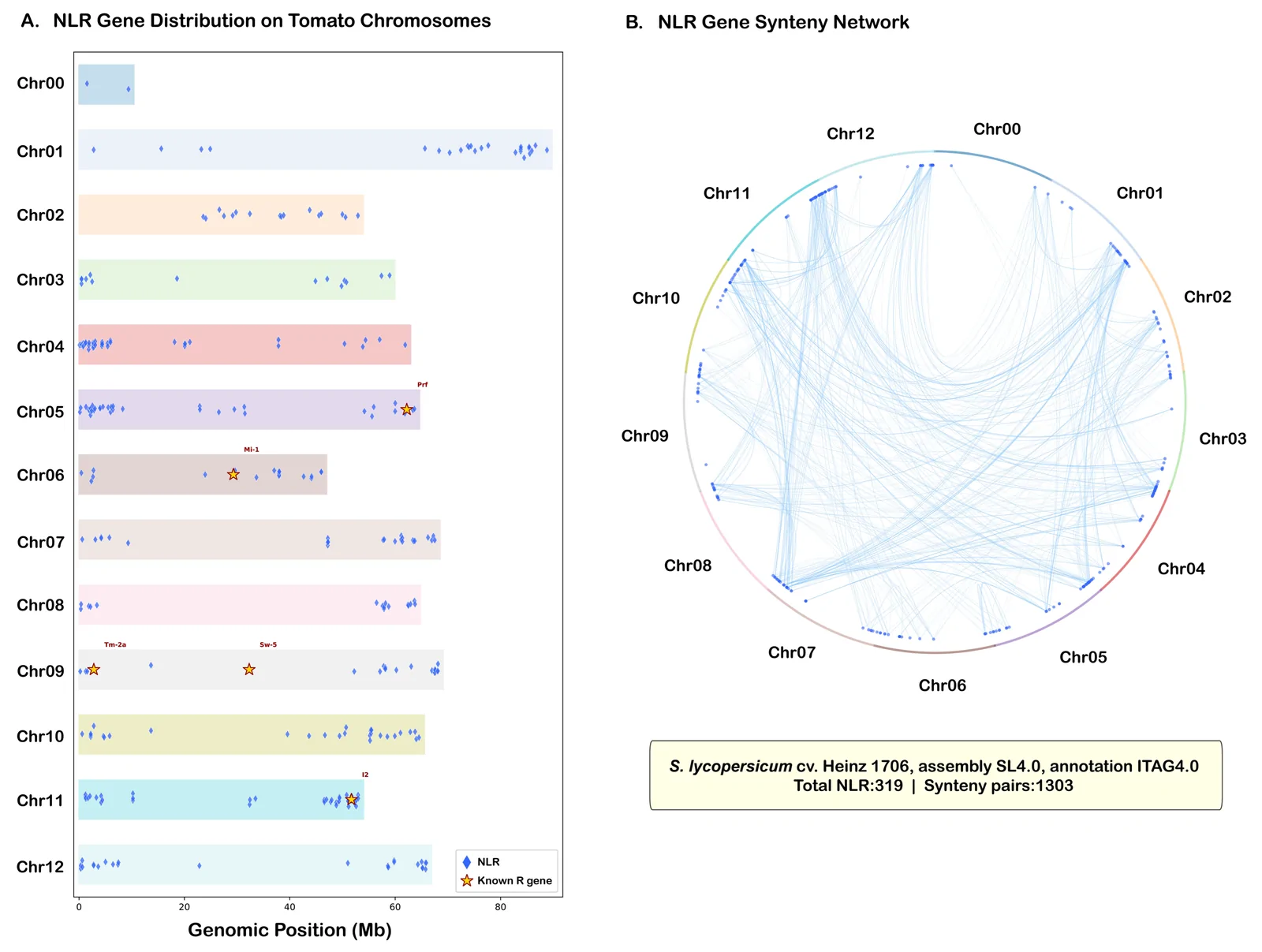
Genomic distribution and syntenic organization of NLR genes in cultivated tomato (*S. lycopersicum*, assembly SL4.0). (**A**) Circos plot illustrating the chromosomal distribution of 319 NLR genes across the 12 tomato chromosomes. The inner histogram track displays NLR gene density calculated in 1 Mb sliding windows, highlighting non-uniform distribution with prominent clusters in pericentromeric and distal chromosomal regions. (**B**) Synteny network of tomato NLR genes. Each node represents an individual NLR locus; edges connect gene pairs residing within the same collinear block. A total of 1303 syntenic NLR gene pairs were identified, with hub nodes of high connectivity corresponding to large, tandemly arranged NLR clusters. Multiple inter-chromosomal connections reflect the contribution of ancestral whole-genome duplication and segmental duplication events to the current NLR genomic architecture.

NLR genes were non-uniformly distributed across the 12 chromosomes of cultivated tomato (Figure 1A). Prominent NLR-dense clusters were evident on multiple chromosomes, particularly in pericentromeric and distal chromosomal regions, while other chromosomes harbored comparatively few NLR loci. This non-random distribution is consistent with a history of localized gene duplication events interspersed with large-scale genomic dispersal.

Synteny analysis using MCScanX identified 1303 NLR-related collinear gene pairs in the tomato genome (Figure 1B; Table S2). The resulting collinearity network revealed extensive syntenic relationships among NLR loci, with multiple high-connectivity hub nodes corresponding to large, tandemly arranged NLR gene clusters. Notably, numerous collinear connections spanning non-homologous chromosomes were detected, implicating ancestral whole-genome duplication (WGD) or segmental duplication events in shaping the current NLR repertoire. The coexistence of local (tandem) and distal (segmental) collinear connections reflects the complex history of duplication and rearrangement that has sculpted the genomic architecture of tomato NLR genes.

### 3.3. The Comparative Pan-NLRome Exhibits a Core–Shell–Cloud Structure

**Figure 2:**
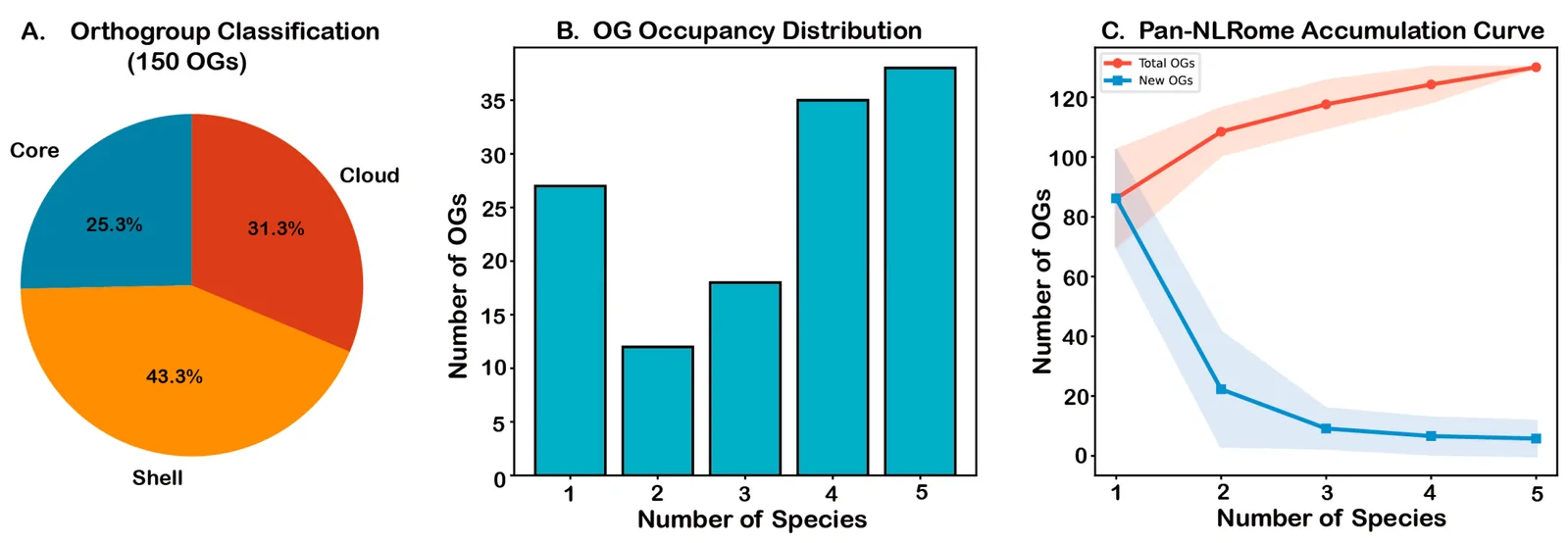
OG classification and pan-NLRome structure across five angiosperm species. (**A**) Classification of 150 NLR OGs into Core (present in all five species; *n* = 38, 25.3%), Shell (present in 2–4 species; *n* = 65, 43.3%), and Cloud (species-specific; *n* = 47, 31.3%) categories, shown by both OG count and proportional gene content. (**B**) Species-occupancy distribution of the 150 OGs, revealing a characteristic U-shaped pattern with peaks at both the lowest (one species) and highest (five species) occupancy extremes. This bimodal distribution reflects the coexistence of a broadly conserved core and a highly variable, species-specific periphery within the pan-NLRome. (**C**) Pan-NLRome accumulation curves depicting the cumulative number of OGs (upper curve) and the number of newly discovered OGs (lower curve) as species are sequentially incorporated. Curves were generated from 100 random species-inclusion permutations; shaded areas represent 95% confidence intervals. The consistently positive slope of the novel OG curve across all permutations confirms the open structure of the tomato pan-NLRome.

OrthoFinder assigned 1436 genes (91.7% of the 1566 chromosome-anchored NLR genes) to 150 OGs (Figure 2A). Based on species occupancy, these OGs were classified into three pan-NLRome categories: core, shell, and cloud OGs.

The distribution of OGs across categories was markedly uneven. Core OGs numbered only 38 (25.3% of all OGs), while shell OGs (65; 43.3%) and cloud OGs (47; 31.3%) together constituted the majority of OG diversity (Figure 2A). Nevertheless, core OGs consistently harbored larger gene families and therefore contributed disproportionately to the total NLR gene count.

The species-occupancy distribution of OGs followed a characteristic U-shaped pattern, with peaks at both the lowest and highest occupancy extremes (Figure 2B). Cumulative pan-NLRome analysis further confirmed that novel OGs continued to accumulate with each additional species across 100 random species-inclusion permutations (mean ± 95% CI shown in Figure 2C). This monotonically increasing trend establishes that the pan-NLRome has an open structure and implies that the five species examined here are insufficient to capture the full breadth of NLR diversity within Solanaceae.

### 3.4. Phylogenetic Analysis Reveals Two Major Lineage-Specific Radiations

**Figure 3:**
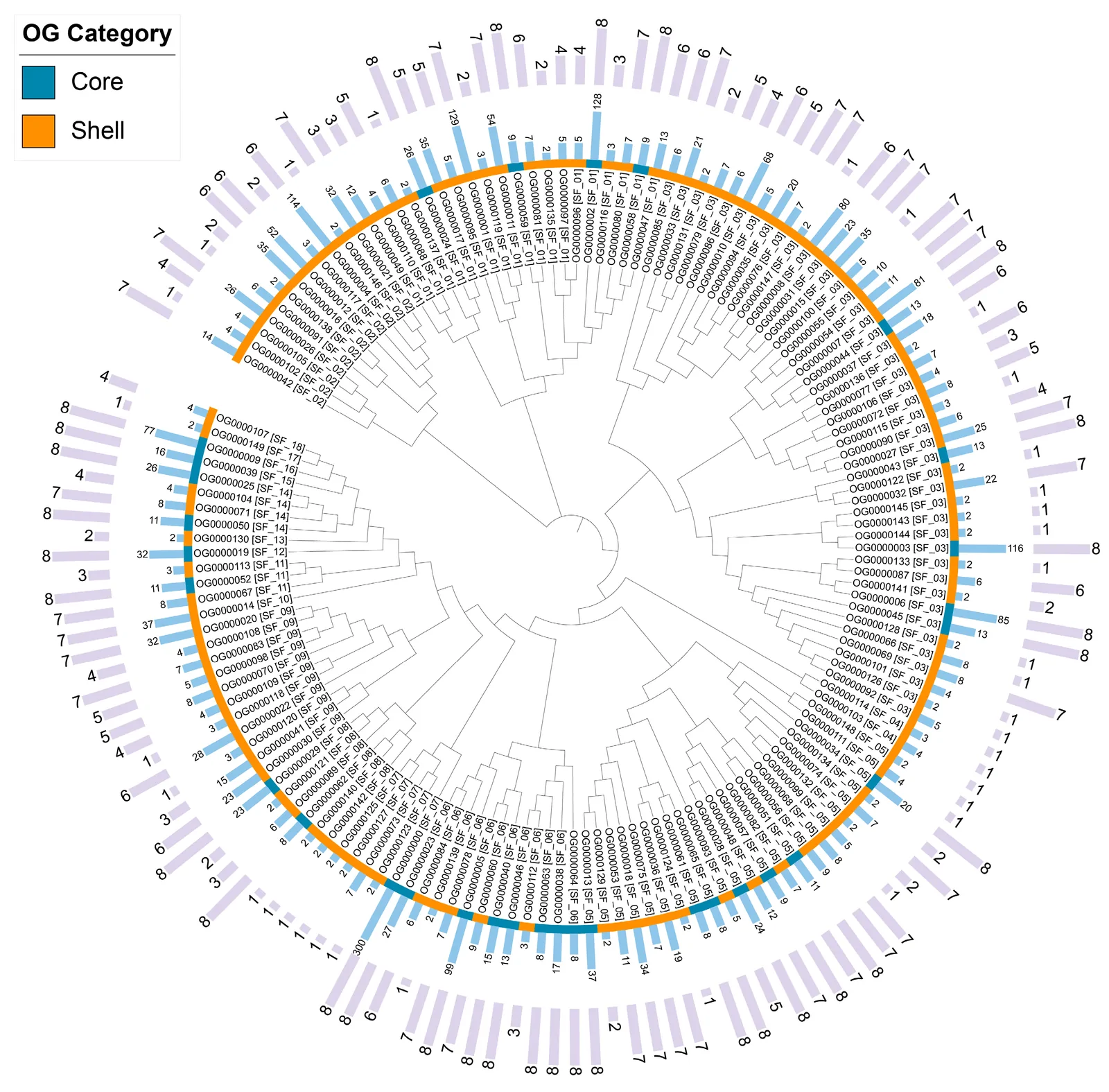
Maximum likelihood phylogenetic tree of the tomato pan-NLRome. The tree was reconstructed from representative protein sequences of all 150 OGs using FastTree v2.1.11 under the WAG+GAMMA substitution model, following multiple sequence alignment with MAFFT and trimming with trimAl v1.4. Eighteen subfamilies (SF_01–SF_18) are delimited by colored clades and labeled accordingly. Three concentric annotation layers are displayed outward from the tree: (i) Core/Shell/Cloud classification of each OG, color-coded to indicate species occupancy breadth; (ii) OG size, represented as a scaled bar proportional to the number of member genes; and (iii) species occupancy, shown as a heatmap indicating the number of species in which each OG is present. Branch support values ≥ 0.90 (local support) are marked at internal nodes. The two most expanded subfamilies, SF_03 and SF_01, are highlighted, jointly accounting for approximately 79% of all chromosomally anchored NLR genes. Shell and Cloud OGs are predominantly enriched at terminal branches, consistent with recent lineage-specific diversification events.

A maximum likelihood phylogenetic tree was constructed from representative sequences of all 150 OGs (Figure 3). Integrating phylogenetic topology, branch support values, and OG clustering patterns, the pan-NLRome was partitioned into 18 subfamilies (SF_01–SF_18; Table 2). Gene content was highly unequal across subfamilies: SF_03 and SF_01 were by far the most expanded, together dominating the overall phylogenetic landscape. SF_03 encompassed 771 genes across 44 OGs, and SF_01 contained 464 genes across 20 OGs; these two lineages jointly accounted for approximately 79.0% of the entire NLR gene pool (1235/1566 = 78.9%) and formed large, well-supported independent clades in the phylogenetic tree.

Notably, several of the largest OGs by gene copy number belonged to the shell category rather than the core. This indicates that certain NLR lineages have undergone pronounced species-specific expansion without achieving broad phylogenetic conservation across the five species. SF_07 exemplifies this pattern: OGs within this clade averaged 62.6 genes per group yet occurred in only 2.4 species on average (Table 2), reflecting extensive gene amplification confined to a narrow phylogenetic range. These findings reveal a partial decoupling between gene copy number and phylogenetic conservation within the NLR superfamily—the most highly amplified lineages are not necessarily the most broadly conserved.

Annotation layers on the phylogenetic tree further showed that core OGs were distributed broadly across the tree, whereas shell and cloud OGs were enriched in multiple terminal branches, suggesting recent lineage-specific diversification events (Figure 3). Accompanying bar chart annotations highlighted marked heterogeneity in OG size and species distribution across lineages, with OGs exhibiting high copy number but limited species range concentrated in specific clades—indicative of asymmetric amplification among evolutionarily dynamic NLR lineages. The remaining 16 subfamilies were substantially smaller, typically comprising only a handful of OGs and genes. This pronounced asymmetry reinforces the conclusion that evolutionary amplification across NLR lineages is fundamentally unbalanced.

### 3.5. Spatially Dispersed Organization Is the Predominant Architectural Pattern in the Pan-NLRome

**Figure 4:**
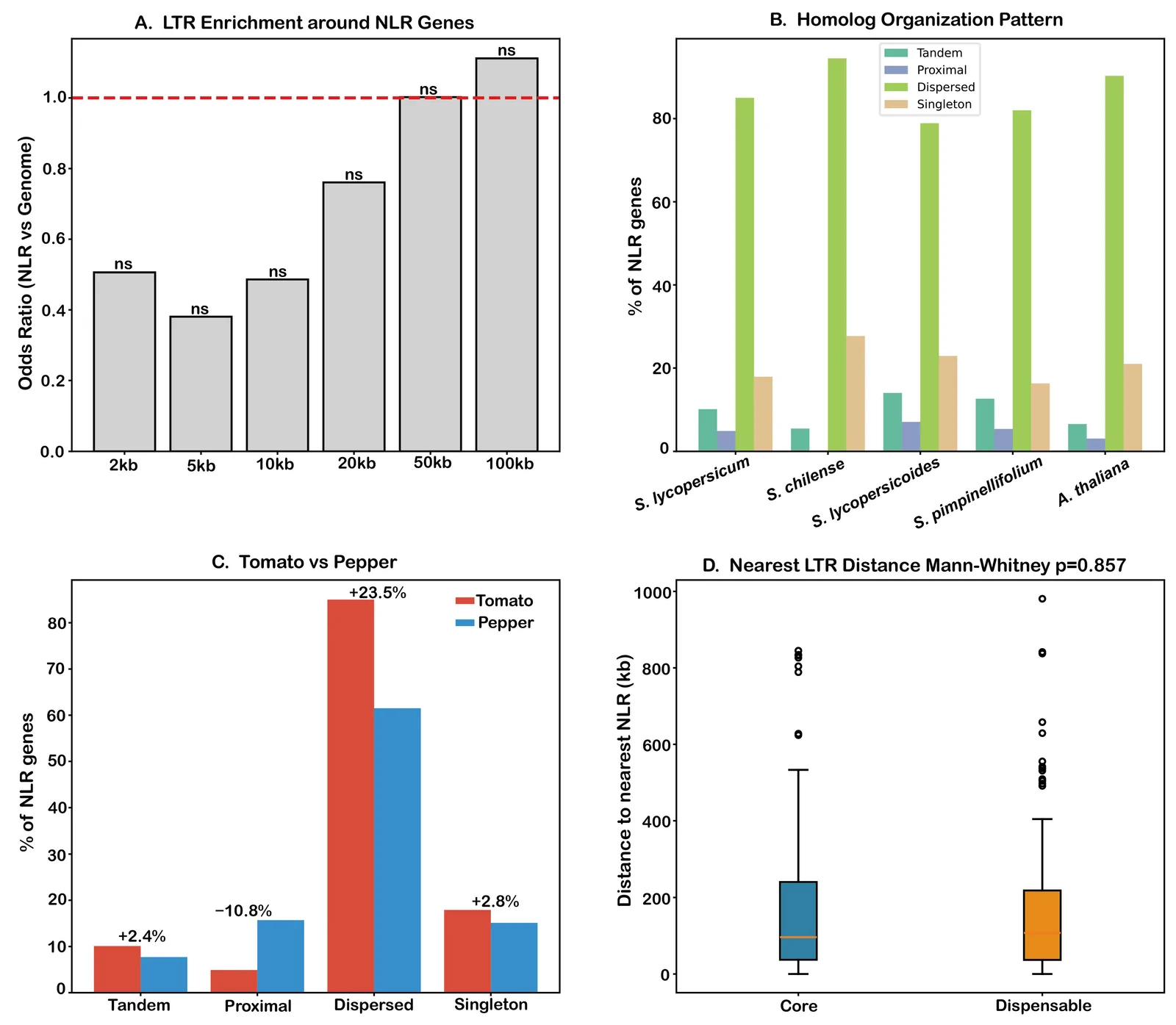
Genomic organization and transposable element associations of NLR genes in cultivated tomato and related species. (**A**) Enrichment analysis of full-length LTR retrotransposons within flanking genomic windows of increasing size (±2, ±5, ±10, ±20, ±50, and ±100 kb) around NLR gene transcription start sites. Odds ratios and *p*-values were derived from Fisher’s exact test, “ns” denotes not significant (p > 0.05), with background rates estimated from 1000 random genomic samplings of equivalent window size. The red dashed line at odds ratio = 1 represents no enrichment. No statistically significant enrichment was detected at any window size (all *p* > 0.05), although odds ratios showed a modest upward trend with increasing window size. (**B**) Stacked bar chart showing the proportions of four spatial organization categories—tandem, proximal, dispersed, and singleton—for NLR homologs across the five analyzed species. Dispersed arrangements are overwhelmingly predominant in all four Solanaceae species, averaging 85.1% of gene pairs. (**C**) Comparison of NLR homolog organization patterns between Solanaceae species (this study) and pepper (*C. annuum*; data from Seo et al. [15]). The substantially lower dispersed proportion in pepper (61.5%) relative to tomato and its relatives highlights a distinctive organizational divergence within Solanaceae. (**D**) Box plots comparing the distance from NLR genes to the nearest full-length LTR retrotransposon between Core and Dispensable (Shell + Cloud) NLR gene sets. Boxes represent the interquartile range (IQR), with the median indicated by a horizontal line. Whiskers extend to 1.5 × IQR; individual points beyond whiskers are shown as outliers. Core NLRs are colored blue and Dispensable NLRs orange. Although Dispensable NLRs showed numerically shorter median distances, the difference was not statistically significant (Mann–Whitney U test, *p* = 0.857).

The most striking organizational feature of the tomato pan-NLRome is the overwhelming prevalence of non-clustered, spatially dispersed homologs across all four Solanaceae species analyzed (Figure 4B; Table 3). On average, 85.1% of NLR genes in Solanaceae were classified as dispersed, far exceeding the proportions of tandem (10.6%) and proximal (4.3%) arrangements. Singleton genes constituted only a minor fraction of the total gene pool. This organizational profile differs markedly from that reported for pepper (*C. annuum*) by Seo et al. [15], in which dispersed homologs accounted for only 61.5% of the NLR repertoire, while tandem (25.2%) and proximal (13.3%) arrangements were substantially more prevalent (Figure 4C). The considerably higher dispersal rate in tomato and its wild relatives suggests that genomic dispersal, rather than local clustering, is the dominant mechanism governing NLR homolog distribution in this lineage.

### 3.6. Spatial Association Between NLR Genes and LTR Retrotransposons Shows No Significant Enrichment

To explore potential genomic factors underlying the dispersed organization of NLR homologs, we examined the spatial relationship between NLR genes and full-length LTR-RTs in the cultivated tomato genome. Across all tested window sizes (±2 kb to ±100 kb), Fisher’s exact test revealed no statistically significant enrichment of LTR-RTs in the vicinity of NLR genes relative to randomly sampled genomic background regions (all comparisons *p* > 0.05; Figure 4A; Table S1). Nevertheless, the odds ratios exhibited a consistent upward trend with increasing window size, suggesting a weak spatial association that becomes more apparent at broader genomic scales. We further compared the proximity of core versus non-core (Shell + Cloud) NLR genes to their nearest full-length LTR-RT (Figure 4D). Numerically, non-core NLR genes had shorter median distances to adjacent LTR-RTs than core NLR genes, though this difference did not reach statistical significance (Mann–Whitney U test, *p* = 0.857). While these results do not provide statistically robust support, the directional trend—whereby evolutionarily dynamic, non-core NLR loci tend to reside in closer proximity to LTR-RTs—is broadly consistent with the hypothesis that actively evolving NLR loci preferentially occupy transposon-rich genomic neighborhoods. However, given the absence of significant enrichment across all analytical windows, this interpretation remains speculative and warrants validation through more targeted or expanded analyses.

## 4. Discussion

### 4.1. The Comparative Pan-NLRome Is Predominantly Open and Species-Specific

In this study, we established a pan-NLRome framework spanning five angiosperm species, with cultivated tomato (*S. lycopersicum*) as the focal reference. A total of 1566 chromosome-anchored NLR genes were identified and assigned to 150 OGs. Cumulative analysis demonstrated that novel OGs continued to emerge with each additional species included, confirming that the tomato pan-NLRome has an open structure and that the current five-species sampling has not yet captured the full breadth of NLR diversity in Solanaceae. This finding is consistent with the open pan-NLRome reported in *A. thaliana*, where most NLR OGs exhibit low occupancy frequency and pronounced genotype specificity, and the total OG count continues to grow as additional accessions are incorporated [33]. Analogous patterns have been documented in wild and cultivated barley (*Hordeum vulgare*), where pan-genomic analyses revealed substantial variation in NLR gene content across accessions, with a large proportion of NLR loci absent from any single reference genome [34]. The open structure of the tomato pan-NLRome implies that even the widely used Heinz 1706 reference assembly captures only a fraction of the NLR diversity present across Solanaceae. It should be noted that the cross-species framework employed here differs conceptually from the within-species pan-NLRome approach used in Arabidopsis research [4], which is designed to characterize allelic and structural variation among accessions of the same species.

Although the HMM+BLASTp strategy used here enables consistent identification of intact protein-coding NLR genes across multiple Solanum genomes, it is dependent on the quality of available genome annotations. Therefore, atypical NLRs, partial NLR fragments, and pseudogene-like loci lacking complete open reading frames may not be fully captured. Genome-based annotation-independent tools, such as NLR-Annotator [35] and Resistify, provide complementary approaches for identifying additional NLR-related loci directly from genome assemblies. Future integration of these approaches will help further refine the pan-NLRome landscape and distinguish annotation-associated differences from true evolutionary variation.

The core–shell–cloud classification further revealed that core OGs—those present in all five species—accounted for only 25.3% of total OG diversity yet contributed disproportionately to overall NLR gene content owing to their larger average family size. This pattern mirrors observations from wheat (*Triticum aestivum*) pan-genome studies, in which conserved NLR lineages tend to be ancient, gene-rich families, whereas species- or accession-specific NLR loci are typically small and of more recent origin [35]. The U-shaped species-occupancy distribution observed here—with peaks at both distribution extremes—points to a bimodal evolutionary architecture: a conserved core maintained by purifying selection, and a rapidly diversifying periphery driven by ongoing co-evolution with pathogen effector repertoires [4].

### 4.2. Chromosomal Distribution and Syntenic Architecture Reflect a Complex Duplication History

NLR genes were non-uniformly distributed across the 12 tomato chromosomes, forming conspicuous clusters in pericentromeric regions and chromosomal distal ends. Such non-random distribution is a hallmark of NLR gene families across diverse plant lineages. In rice (*Oryza sativa*), NLR genes are similarly concentrated in gene-dense clusters at chromosomal ends and enriched in pericentromeric regions [36], while in Arabidopsis, NLR clusters preferentially occupy heterochromatin–euchromatin boundary zones flanking centromeres [33]. The distribution pattern observed in tomato is thus consistent with broadly conserved chromosomal organizational features, likely reflecting shared constraints imposed by recombination suppression and chromatin accessibility.

Synteny analysis identified 1303 NLR-related collinear gene pairs, with numerous connections spanning non-homologous chromosomes—a pattern consistent with the known history of whole-genome duplication (WGD) and segmental duplication in Solanaceae. The tomato genome has undergone at least a Solanaceae-shared triplication event, as well as an older polyploidization event common to all eudicots [19]. Collinear NLR gene pairs originating from WGD are expected to reside on non-homologous chromosomes and to be more sequence-divergent than tandemly duplicated paralogs—a pattern that aligns well with the predominance of dispersed homologs observed in this study. Collectively, the chromosomal distribution and syntenic architecture indicate that the tomato NLR gene pool has been jointly shaped by local tandem duplication and large-scale genomic rearrangement.

### 4.3. Dispersed Homolog Architecture Is a Distinguishing Feature of the Solanaceae NLR Repertoire

The most prominent organizational feature identified in this study is the overwhelming dominance of spatially dispersed NLR homologs across all four Solanaceae species analyzed. On average, 85.1% of NLR gene pairs in Solanaceae were classified as dispersed—substantially higher than the 61.5% reported for pepper (*C. annuum*) by Seo et al. [15]. Tandem and proximal configurations, which are prevalent in many other plant lineages, were comparatively rare across the Solanaceae species examined here. This high degree of dispersal stands in contrast to NLR organization patterns reported in several cereal crops. In wheat, approximately 50% of NLR genes occur in tandem or proximal clusters, many of which correspond to functionally characterized disease resistance loci. In maize (*Zea mays*), clustered NLR configurations are similarly associated with broad-spectrum resistance. The prevalence of dispersed NLR organization in tomato and its relatives may therefore reflect lineage-specific evolutionary constraints on recombination-driven diversification, potentially linked to the larger genome size and higher repeat content of Solanaceae compared to Arabidopsis.

The quantitative difference in dispersal ratios between this study and the pepper dataset reported by Seo et al. warrants careful interpretation. That study employed an earlier version of the CM334 genome annotation and different criteria for homolog classification [15]; consequently, methodological differences in window size definitions, intervening-gene thresholds, and OG assignment approaches may introduce quantitative biases. Nevertheless, the directional contrast between tomato and pepper appears biologically robust. Pepper has undergone extensive chromosomal rearrangements, and its genome (~3.5 Gb) is considerably larger than that of tomato (~900 Mb) [37], potentially promoting local NLR clustering through distinct mechanisms. The high dispersal ratio of the tomato pan-NLRome is also consistent with the genomic configurations of several functionally characterized tomato resistance genes—including I-2, Cf-4, and Mi-1.2—which occur as isolated loci or small clusters rather than as members of large tandem arrays [38]. This dispersed genomic landscape is compatible with an evolutionary model in which different NLR loci experience distinct recombination environments and selection pressures. Whether dispersed and clustered NLR loci differ in their rates of recognition specificity evolution, however, remains to be addressed through systematic functional comparative studies.

### 4.4. Asymmetric Subfamily Expansion Reflects Lineage-Specific Adaptive Radiation

Phylogenetic analysis partitioned the 150 OGs into 18 subfamilies, revealing striking size asymmetry: SF_03 and SF_01 were dramatically expanded, jointly accounting for approximately 79% of all NLR genes. Such pronounced subfamily-level asymmetry is a recognized feature of NLR gene family evolution. In plants such as soybean (*Glycine max*), sorghum (*Sorghum bicolor*) and maize (*Z. mays*), a small number of NLR clades account for the majority of gene copies, with rapid birth-and-death dynamics generating extensive expansions of phylogenetically shallow lineages [39]. Similarly, in potato (*S. tuberosum*), a limited set of NLR subfamilies dominates the disease resistance gene landscape and encodes most functionally characterized R proteins [40].

The enrichment of shell and cloud OGs at terminal phylogenetic branches is consistent with a model in which lineage-specific NLR diversification occurs at the periphery of conserved phylogenetic lineages—a pattern interpreted as evidence of persistent adaptive radiation driven by co-evolutionary selection imposed by pathogen effector diversity [41]. Core OGs, distributed broadly across the phylogeny, likely encode immune receptors with conserved recognition functions maintained across species by purifying selection, possibly responsible for monitoring broadly conserved host targets such as guardees or decoys [42]. By contrast, the expanded shell and cloud OGs may encode more recently evolved receptors with narrow or species-specific recognition spectra, consistent with the rapid co-evolutionary dynamics characteristic of the NLR–effector interface.

### 4.5. LTR Retrotransposons and the Dispersed Organization of Tomato NLRs

To explore potential mechanisms underlying the dispersed NLR architecture, we analyzed the spatial relationship between NLR genes and full-length LTR-RTs in the cultivated tomato genome. Across all tested genomic windows (±2 kb to ±100 kb), no statistically significant enrichment of LTR-RTs in the vicinity of NLR genes was detected relative to randomly sampled genomic background regions (all comparisons *p* > 0.05). Similarly, no significant difference in proximity to the nearest full-length LTR-RT was observed between core and non-core NLR genes (Mann–Whitney U test, *p* = 0.857). These null results contrast with findings from other plant systems. In barley, LTR-RTs are significantly enriched near NLR gene clusters, and TE insertions have been proposed to facilitate NLR gene movement and diversification [34]. In flax (Linum usitatissimum), TE-rich regions are similarly associated with elevated NLR gene density. Several factors may account for the absence of a significant LTR-RT enrichment signal in cultivated tomato.

First, the present analysis was restricted to full-length LTR-RTs. The majority of LTR retrotransposons in the tomato genome exist as fragmented or solo LTRs generated by intra-element recombination—a process that eliminates structural integrity while leaving a genomic footprint [19]. These degraded TE-derived sequences, rather than intact LTR-RTs, may constitute the primary transposable element environment influencing NLR dispersal. Future analyses should therefore incorporate the full spectrum of detectable TE-derived sequences, including solo LTRs and truncated elements. Second, the relatively limited number of non-core NLR genes in this study—drawn from 150 OGs across five species—may have constrained statistical power. Pan-NLRome analyses encompassing larger species sets, or population-level NLR diversity datasets, would provide greater resolution for detecting subtle associations between TE content and NLR genomic context [33]. Third, the dispersed organization of tomato NLR genes may be driven primarily by mechanisms other than TE-mediated transposition, such as chromosomal rearrangements associated with ancient polyploidization, ectopic recombination among dispersed paralogs, or purifying selection against large tandem NLR arrays due to the elevated autoimmunity risk they confer [43]. Notably, foundational genome-wide surveys in Arabidopsis demonstrated that NLR genes are frequently organized in clustered arrays yet undergo rapid evolutionary turnover [44], highlighting the need to distinguish between physical clustering and functional linkage when interpreting genomic organization patterns. These alternative mechanisms are not mutually exclusive with TE-mediated effects and may act synergistically over evolutionary time.

Despite the absence of statistical significance, the directional trends observed remain broadly consistent with the hypothesis that evolutionarily dynamic NLR loci preferentially occupy TE-rich genomic neighborhoods. This interpretation is, however, preliminary. Rigorous evaluation of the contribution of transposable elements to NLR dispersal and diversification in Solanaceae will require expanded taxonomic sampling, inclusion of degraded TE-derived sequences, and the integration of population-level NLR variation data.

## 5. Conclusions

This study presents the first comprehensive pan-NLRome analysis spanning cultivated tomato and four closely related species, systematically characterizing the diversity, phylogenetic structure, spatial organization, and genomic context of NLR genes across Solanaceae. The principal findings are summarized below. The tomato pan-NLRome exhibits an open structure: novel OGs continued to accumulate with each additional species included, demonstrating that the full complement of NLR diversity in Solanaceae remains incompletely sampled. This finding underscores the necessity of multi-genome and population-level approaches for comprehensive characterization of crop immune gene repertoires. The pan-NLRome is composed of a conserved core and a large, highly variable periphery. Core OGs constituted only 25.3% of all OGs yet contributed the majority of NLR gene copies, reflecting strong purifying selection on conserved immune lineages. Shell and cloud OGs, enriched at terminal phylogenetic branches, likely encode receptors of more recent origin or narrower phylogenetic distribution, shaped by persistent co-evolutionary pressures imposed by pathogen effector diversity. Spatially dispersed homolog organization is the dominant architectural pattern across all Solanaceae species examined. On average, 85.1% of NLR gene pairs were classified as dispersed—significantly exceeding the proportion reported for pepper—indicating that interchromosomal dispersal, rather than local tandem clustering, is the predominant force shaping the Solanaceae NLR gene pool. This dispersed genomic landscape has important practical implications for the discovery and breeding deployment of novel disease resistance genes.

Phylogenetic analysis revealed substantial asymmetry among NLR subfamilies, highlighting lineage-specific expansion and diversification during tomato immune receptor evolution. In addition, no significant spatial association was detected between NLR genes and full-length LTR retrotransposons in cultivated tomato, suggesting that factors beyond intact LTR proximity may contribute to NLR genome organization. Together, this study provides a comparative genomic framework for understanding NLR diversity and evolution in tomato and related Solanum species.

## Figures and Tables

**Table 1:** Summary of NLR gene identification across eight angiosperm species.

Species	Common Name	NLR Candidates
*S. lycopersicum* (cv. Heinz 1706)	Cultivated tomato	319
*S. chilense*	Wild tomato	206
*S. habrochaites* LA1353	Wild tomato	234
*S. lycopersicoides* LA2951	Wild tomato	513
*S. pimpinellifolium* LA1589	Wild tomato	346
*S. melongena*	Eggplant	388
*S. tuberosum*	Potato	673
*A. thaliana* (TAIR10)	Arabidopsis	300

Note: *S. habrochaites*, *S. melongena*, and *S. tuberosum* (shaded) were excluded from all downstream comparative analyses owing to annotation structure incompatibilities that precluded reliable chromosomal anchoring. The five retained species collectively yielded 1566 chromosomally anchored NLR genes.

**Table 2:** Subfamily classification of the tomato Pan-NLRome (18 subfamilies).

Subfamily	OG Count	Avg Species Occupancy	Total Genes	Avg Genes per OG
SF_03	44	4.5	771	17.5
SF_01	20	5.1	464	23.2
SF_07	5	2.4	313	62.6
SF_02	12	4.2	294	24.5
SF_05	24	5.6	255	10.6
SF_06	12	6.7	214	17.8
SF_09	11	4.5	132	12.0
SF_16	1	8.0	77	77.0
SF_14	4	6.8	49	12.2
SF_08	6	3.8	43	7.2
SF_10	1	7.0	37	37.0
SF_12	1	8.0	32	32.0
SF_11	3	6.0	22	7.3
SF_15	1	8.0	16	16.0
SF_04	2	1.0	7	3.5
SF_18	1	4.0	4	4.0
SF_13	1	2.0	2	2.0
SF_17	1	1.0	2	2.0
Total	150	—	2734	—

Note: The header “Avg” in the third and fifth columns is an abbreviation for “Average”. The total of 2734 NLR genes represents the initial OG classification dataset derived from eight screened genome resources. The pan-NLRome analyses presented in this study were performed using 1566 chromosome-anchored NLR genes from five species retained after genome assembly and annotation quality filtering.

**Table 3:** Spatial organization of NLR homologs across five analyzed species.

Species	Label	Total NLR	Total Pairs	Tandem	Proximal	Dispersed	Singleton	Tandem (%)	Proximal (%)	Dispersed (%)	Singleton (%)
*S. lycopersicum*	Cultivated tomato	319	1303	132	64	1107	57	10.1	4.9	85.0	17.9
*S. chilense*	Wild tomato	206	542	30	0	512	57	5.5	0	94.5	27.7
*S. lycopersicoides*	Wild tomato	459	2827	396	201	2230	105	14.0	7.1	78.9	22.9
*S. pimpinellifolium*	Wild tomato	344	1502	189	81	1232	56	12.6	5.4	82.0	16.3
*A. thaliana*	Arabidopsis	238	2061	135	64	1862	50	6.6	3.1	90.3	21.0
Mean		332	1544	187	87	1270	69	10.6	4.3	85.1	21.2
*C. annuum* *	Pepper	—	—	—	—	—	—	25.2	13.3	61.5	—

* Genomic data of pepper were retrieved from the study published by Seo et al. [15]; absolute gene counts not available for direct comparison. Note: Spatial categories are defined as follows—Tandem: homologous gene pairs separated by ≤200 kb with ≤10 intervening genes; Proximal: separated by ≤200 kb but with >10 intervening genes; Dispersed: located on different chromosomes or separated by >200 kb; Singleton: NLR genes lacking any detectable homologous partner within the same OG. Percentage values for Tandem, Proximal, and Dispersed are calculated relative to total gene pairs; Singleton (%) is calculated relative to total NLR gene count. The Singleton category is not mutually exclusive with the pair-based categories.

## Data Availability

The original contributions presented in this study are included in the article and Supplementary Materials. Further inquiries regarding the data can be directed to the corresponding authors.

## Supplementary Materials

The following supporting information can be downloaded at: https://www.mdpi.com/article/10.3390/genes17080864/s1, Table S1: Full Results of Multi-Window Fisher’s Exact Test for LTR-RTs (Odds Ratios and *p*-values across 6 Windows); Table S2: List of NLR Duplicate Pairs in Cultivated Tomato (1303 Pairs).

## References

[1] Jones J.D.G., Vance R.E., Dangl J.L. (2016). Intracellular innate immune surveillance devices in plants and animals. Science.

[2] Ngou B.P.M., Ding P., Jones J.D.G. (2022). Thirty years of resistance: Zig-zag through the plant immune system. Plant Cell.

[3] Kourelis J., van der Hoorn R.A.L. (2018). Defended to the Nines: 25 Years of Resistance Gene Cloning Identifies Nine Mechanisms for R Protein Function. Plant Cell.

[4] Van de Weyer A.-L., Monteiro F., Furzer O.J., Nishimura M.T., Cevik V., Witek K., Jones J.D.G., Dangl J.L., Weigel D., Bemm F. (2019). A Species-Wide Inventory of NLR Genes and Alleles in *Arabidopsis thaliana*. Cell.

[5] Wang J., Hu M., Wang J., Qi J., Han Z., Wang G., Qi Y., Wang H.-W., Zhou J.-M., Chai J. (2019). Reconstitution and structure of a plant NLR resistosome conferring immunity. Science.

[6] Bi G., Su M., Li N., Liang Y., Dang S., Xu J., Hu M., Wang J., Zou M., Deng Y. (2021). The ZAR1 resistosome is a calcium-permeable channel triggering plant immune signaling. Cell.

[7] Huang S., Jia A., Song W., Hessler G., Meng Y., Sun Y., Xu L., Laessle H., Jirschitzka J., Ma S. (2022). Identification and receptor mechanism of TIR-catalyzed small molecules in plant immunity. Science.

[8] Yu D., Song W., Tan E.Y.J., Liu L., Cao Y., Jirschitzka J., Li E., Logemann E., Xu C., Huang S. (2022). TIR domains of plant immune receptors are 2′,3′-cAMP/cGMP synthetases mediating cell death. Cell.

[9] Lolle S., Stevens D., Coaker G. (2020). Plant NLR-triggered immunity: From receptor activation to downstream signaling. Curr. Opin. Immunol..

[10] Alonge M., Wang X., Benoit M., Soyk S., Pereira L., Zhang L., Suresh H., Ramakrishnan S., Maumus F., Ciren D. (2020). Major Impacts of Widespread Structural Variation on Gene Expression and Crop Improvement in Tomato. Cell.

[11] Della Coletta R., Qiu Y., Ou S., Hufford M.B., Hirsch C.N. (2021). How the pan-genome is changing crop genomics and improvement. Genome Biol..

[12] Bayer P.E., Golicz A.A., Scheben A., Batley J., Edwards D. (2020). Plant pan-genomes are the new reference. Nat. Plants.

[13] Gao L., Gonda I., Sun H., Ma Q., Bao K., Tieman D.M., Burzynski-Chang E.A., Fish T.L., Stromberg K.A., Sacks G.L. (2019). The tomato pan-genome uncovers new genes and a rare allele regulating fruit flavor. Nat. Genet..

[14] Zhou Y., Zhang Z., Bao Z., Li H., Lyu Y., Zan Y., Wu Y., Cheng L., Fang Y., Wu K. (2022). Graph pangenome captures missing heritability and empowers tomato breeding. Nature.

[15] Seo E., Kim S., Yeom S.-I., Choi D. (2016). Genome-Wide Comparative Analyses Reveal the Dynamic Evolution of Nucleotide-Binding Leucine-Rich Repeat Gene Family among Solanaceae Plants. DNA Res..

[16] Mistry J., Chuguransky S., Williams L., Qureshi M., Salazar G.A., Sonnhammer E.L.L., Tosatto S.C.E., Paladin L., Raj S., Richardson L.J. (2020). Pfam: The protein families database in 2021. Nucleic Acids Res..

[17] Eddy S.R. (2011). Accelerated Profile HMM Searches. PLoS Comput. Biol..

[18] Emms D.M., Kelly S. (2019). OrthoFinder: Phylogenetic orthology inference for comparative genomics. Genome Biol..

[19] The Tomato Genome Consortium (2012). The tomato genome sequence provides insights into fleshy fruit evolution. Nature.

[20] Reiser L., Bakker E., Subramaniam S., Chen X., Sawant S., Khosa K., Prithvi T., Berardini T.Z. (2024). The Arabidopsis Information Resource in 2024. Genetics.

[21] Altschul S.F., Madden T.L., Schäffer A.A., Zhang J., Zhang Z., Miller W., Lipman D.J. (1997). Gapped BLAST and PSI-BLAST: A new generation of protein database search programs. Nucleic Acids Res..

[22] Krzywinski M., Schein J., Birol İ., Connors J., Gascoyne R., Horsman D., Jones S.J., Marra M.A. (2009). Circos: An information aesthetic for comparative genomics. Genome Res..

[23] Wang Y., Tang H., DeBarry J.D., Tan X., Li J., Wang X., Lee T.H., Jin H., Marler B., Guo H. (2012). MCScanX: A toolkit for detection and evolutionary analysis of gene synteny and collinearity. Nucleic Acids Res..

[24] Shannon P., Markiel A., Ozier O., Baliga N.S., Wang J.T., Ramage D., Amin N., Schwikowski B., Ideker T. (2003). Cytoscape: A software environment for integrated models of biomolecular interaction networks. Genome Res..

[25] Katoh K., Standley D.M. (2013). MAFFT multiple sequence alignment software version 7: Improvements in performance and usability. Mol. Biol. Evol..

[26] Capella-Gutiérrez S., Silla-Martínez J.M., Gabaldón T. (2009). trimAl: A tool for automated alignment trimming in large-scale phylogenetic analyses. Bioinformatics.

[27] Price M.N., Dehal P.S., Arkin A.P. (2010). FastTree 2—Approximately maximum-likelihood trees for large alignments. PLoS ONE.

[28] Letunic I., Bork P. (2021). Interactive Tree Of Life (iTOL) v5: An online tool for phylogenetic tree display and annotation. Nucleic Acids Res..

[29] Xu Z., Wang H. (2007). LTR_FINDER: An efficient tool for the prediction of full-length LTR retrotransposons. Nucleic Acids Res..

[30] Ellinghaus D., Kurtz S., Willhoeft U. (2008). LTRharvest, an efficient and flexible software for de novo detection of LTR retrotransposons. BMC Bioinform..

[31] Ou S., Jiang N. (2017). LTR_retriever: A Highly Accurate and Sensitive Program for Identification of Long Terminal Repeat Retrotransposons. Plant Physiol..

[32] Benjamini Y., Hochberg Y. (1995). Controlling the False Discovery Rate: A Practical and Powerful Approach to Multiple Testing. J. R. Stat. Soc. Ser. B (Methodol.).

[33] Prigozhin D.M., Krasileva K.V. (2021). Analysis of intraspecies diversity reveals a subset of highly variable plant immune receptors and predicts their binding sites. Plant Cell.

[34] Jayakodi M., Padmarasu S., Haberer G., Bonthala V.S., Gundlach H., Monat C., Lux T., Kamal N., Lang D., Himmelbach A. (2020). The barley pan-genome reveals the hidden legacy of mutation breeding. Nature.

[35] Steuernagel B., Witek K., Krattinger S.G., Ramirez-Gonzalez R.H., Schoonbeek H.-J., Yu G., Baggs E., Witek A.I., Yadav I., Krasileva K.V. (2020). The NLR-Annotator Tool Enables Annotation of the Intracellular Immune Receptor Repertoire. Plant Physiol..

[36] Wang Y., Dorjee T., Wang Y., Peng D., Chen L., Wei C., Sun S., Duan M., Li H., Hathorn A. (2026). Pan-NLRome analysis uncovers genetic diversity and evolutionary dynamics among rice, maize and sorghum. Plant Genome.

[37] Kim S., Park M., Yeom S.-I., Kim Y.-M., Lee J.M., Lee H.-A., Seo E., Choi J., Cheong K., Kim K.-T. (2014). Genome sequence of the hot pepper provides insights into the evolution of pungency in Capsicum species. Nat. Genet..

[38] Milligan S.B., Bodeau J., Yaghoobi J., Kaloshian I., Zabel P., Williamson V.M. (1998). The root knot nematode resistance gene Mi from tomato is a member of the leucine zipper, nucleotide binding, leucine-rich repeat family of plant genes. Plant Cell.

[39] Lozano R., Gazave E., dos Santos J.P.R., Stetter M.G., Valluru R., Bandillo N., Fernandes S.B., Brown P.J., Shakoor N., Mockler T.C. (2021). Comparative evolutionary genetics of deleterious load in sorghum and maize. Nat. Plants.

[40] Jupe F., Pritchard L., Etherington G.J., MacKenzie K., Cock P.J.A., Wright F., Sharma S.K., Bolser D., Bryan G.J., Jones J.D.G. (2012). Identification and localisation of the NB-LRR gene family within the potato genome. BMC Genom..

[41] Barragan A.C., Weigel D. (2020). Plant NLR diversity: The known unknowns of pan-NLRomes. Plant Cell.

[42] van der Hoorn R.A.L., Kamoun S. (2008). From Guard to Decoy: A new model for perception of plant pathogen effectors. Plant Cell.

[43] Alcázar R., García A.V., Parker J.E., Reymond M. (2009). Incremental steps toward incompatibility revealed by Arabidopsis epistatic interactions modulating salicylic acid pathway activation. Proc. Natl. Acad. Sci. USA.

[44] Meyers B.C., Kozik A., Griego A., Kuang H., Michelmore R.W. (2003). Genome-wide analysis of NBS-LRR-encoding genes in Arabidopsis. Plant Cell.

